# Identification and characterization of sugar-regulated promoters in *Chaetomium thermophilum*

**DOI:** 10.1186/s12896-023-00791-9

**Published:** 2023-07-08

**Authors:** Sven Reislöhner, Geza Schermann, Max Kilian, Daniela Santamaría-Muñoz, Christian Zimmerli, Nikola Kellner, Jochen Baßler, Michael Brunner, Ed Hurt

**Affiliations:** 1 Biochemistry Center (BZH), University of Heidelberg, Im Neuenheimer Feld 328, 69120 Heidelberg, Germany; 2 Institute for Neurovascular Cell Biology, University Hospital Bonn, Venusberg-Campus 1, 53127 Bonn, Germany; 3 Max-Planck-Institute für terrestrische Mikrobiologie, Marburg, Germany; 4 Department of Cell Biology and Human Anatomy, University of California, Davis, USA; 5 Department of Molecular Sociology, Max Planck Institute of Biophysics, Max-Von-Laue-Straße 3, Frankfurt Am Main, 60438 Germany

**Keywords:** Eukaryotic thermophiles, Filamentous fungi, Inducible promoters, Thermostable proteins, Transcriptomes

## Abstract

The thermophilic fungus *Chaetomium thermophilum* has been used extensively for biochemical and high-resolution structural studies of protein complexes. However, subsequent functional analyses of these assemblies have been hindered owing to the lack of genetic tools compatible with this thermophile, which are typically suited to other mesophilic eukaryotic model organisms, in particular the yeast *Saccharomyces cerevisiae*. Hence, we aimed to find genes from *C. thermophilum* that are expressed under the control of different sugars and examine their associated 5’ untranslated regions as promoters responsible for sugar-regulated gene expression. To identify sugar-regulated promoters in *C. thermophilum,* we performed comparative xylose- versus glucose-dependent gene expression studies, which uncovered a number of enzymes with induced expression in the presence of xylose but repressed expression in glucose-supplemented media. Subsequently, we cloned the promoters of the two most stringently regulated genes, the xylosidase-like gene (*XYL*) and xylitol dehydrogenase (*XDH*), obtained from this genome-wide analysis in front of a thermostable yellow fluorescent protein (YFP) reporter. With this, we demonstrated xylose-dependent YFP expression by both Western blotting and live-cell imaging fluorescence microscopy. Prompted by these results, we expressed the *C. thermophilum* orthologue of a well-characterized dominant-negative ribosome assembly factor mutant, under the control of the *XDH* promoter, which allowed us to induce a nuclear export defect on the pre-60S subunit when *C. thermophilum* cells were grown in xylose- but not glucose-containing medium. Altogether, our study identified xylose-regulatable promoters in *C. thermophilum*, which might facilitate functional studies of genes of interest in this thermophilic eukaryotic model organism.

**Supplementary Information:**

The online version contains supplementary material available at 10.1186/s12896-023-00791-9.

## Background

The proteins of thermophilic microorganisms are of interest for their superior stabilities, both in isolation and in protein complexes, compared to their mesophilic counterparts [1, 2]. A prominent example of their utility is that of the Taq polymerase from the bacterium *Thermus aquaticus* on which the classical PCR method relies [3, 4]. In contrast to bacteria and archaea, thermophily is a rare phenomenon in eukaryotes [5–7]. One such eukaryotic thermophile is the filamentous ascomycete *Chaetomium thermophilum*, which optimally thrives at temperatures between 50–55 °C, with an upper limit of approximately 60 °C [8, 9]. Its genome was sequenced and published in 2011 [8, 10], which finally allowed to reconstitute the assembly and the structure of large nucleoporins of the nuclear pore complex [8, 11, 12]. Along that line, also the reconstitution of protein complexes involved in ribosome assembly or their structural analysis has been successfully studied using its thermostable proteins [13–17]. Moreover, also several other structural studies benefitted from its recombinant thermostable proteins [18–20] and protein complexes, like the huge 600 MDa INO80 complex in contact with the nucleosome [21], showing close insights into structural arrangements. Other large assemblies of several proteins, like the fatty acid synthetase were isolated from wildtype *C. thermophilum* extracts by size exclusion chromatography (SEC) and structurally characterized [22]. Besides for fundamental research, heterologous expression of its thermostable proteins was also successfully applied for industrial purposes in already established mesophilic filamentous fungi, like in *Trichoderma reesei*. Here, the cellulase coding *cbh* gene from the thermophile showed higher cellulase activity and cellulase secretion than the endogenous enzyme in *T. reesei *[23]*.* Moreover, *C. thermophilum* was exploited as a native source for secreted thermostable lignocellulolytic enzymes and their potential for the generation of biofuel out of lignocellulose [24].

To further exploit *C. thermophilum* for structural biology studies and biotechnology, our lab aimed to develop genetic tools to establish *C. thermophilum* as a novel model organism. We have previously published a transformation protocol [25] that allows affinity-tagged proteins and complexes to be purified directly from *C. thermophilum* for biochemical and structural studies. Based on this milestone achievement, cryo-EM studies have afforded various unprecedented structural insights especially along the early ribosomal maturation steps [15, 26, 27].

However, a highly useful, but as yet unavailable, genetic tool would be an inducible promoter that facilitates regulated gene expression in *C. thermophilum*. A broad collection of regulatable promoters in fungi have been described that react to inducers as diverse as various carbon- and nitrogen-based nutrients, metal ions, and even light [28–31]. Other successfully applied promoters are synthetic systems, for example, the Tet On/Off system [32], which is commonly used in the *Aspergillus* genus [33, 34].

Glucose-repressed promoters that can be activated by alternative host-specific carbon sources are commonly applied in filamentous fungi. Among these are the *cellobiohydrolase 1* (*cbh1)* promoter in *Trichoderma reesei* that is activated by a broad range of hemicellulose-derived carbon sources [23, 35]*,* the alcohol dehydrogenase (*alcA)* promoter in *Aspergillus nidulans* that is activated by ethanol [36, 37], and the dehydroquinase promoter (*qa-2*) in *Neurospora crassa* that is activated by quinic acid [38].

Here, we aimed to find sugar-controlled genes in the genome of *C. thermophilum* to evaluate the associated 5’ untranslated regions as promoters for sugar-regulated gene expression. To this end, we grew *C. thermophilum* in minimal media designed to provide either glucose or xylose as the sole carbon source, and analyzed the respective transcriptomes by Illumina deep sequencing. This transcriptome analysis allowed us to identify exclusively active genes in glucose or xylose, respectively. We verified the transcriptomic dynamics for selected genes by quantitative real-time PCR (qRT-PCR). Furthermore, we used a thermostable YFP-reporter construct to monitor protein expression under the control of various promoters by immunoblotting and fluorescence microscopy. Using these approaches, we identified the promoters from a *β*-xylosidase-like gene (*XYL*) and xylitol dehydrogenase (*XDH*) as the most stringently regulated. Both promoters enabled regulated expression of proteins of interest in *C. thermophilum* and will be utilized in future in vivo studies.

## Results

### Establishing sugar-specific media suitable for growing C. thermophilum

**Fig. 1:**
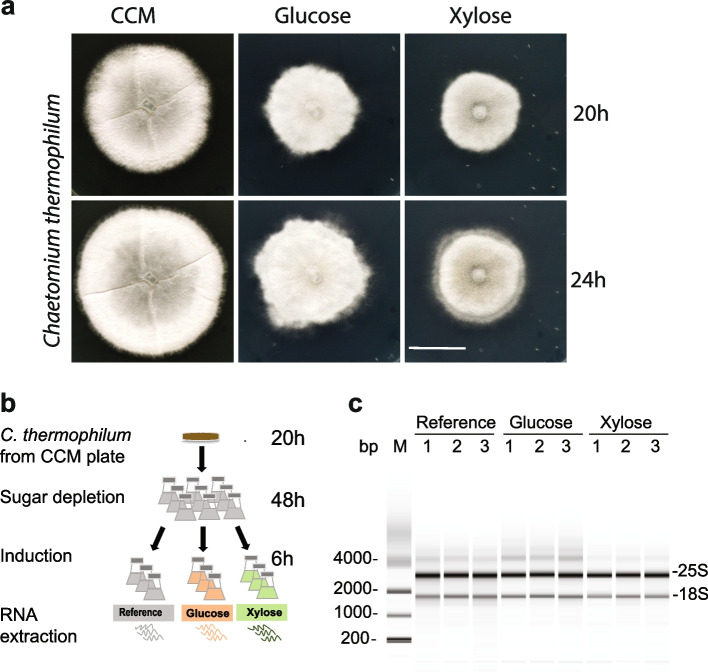
RNA isolation from *C. thermophilum* cultures grown in glucose and xylose. **a** *C. thermophilum* was grown on a traditional CCM plate for 24 h at 50 °C. From a parental colony grown on a CCM plate, equally sized (approximately 3 mm^2^) pieces were excised from the periphery and transferred onto fresh plates with either CCM (control medium), glucose-containing medium, or xylose-containing medium. Colony growth was imaged after 20 and 24 h of incubation at 50 °C. Scale bar: 2 cm. **b** A schematic workflow illustrating the steps of *C. thermophilum* cultivation prior to RNA extraction. A piece of a parent colony was used to inoculate a liquid SPY culture, which was then incubated for 48 h at 55 °C to deplete a putative cellular sugar reserve. Final cultivation was done in liquid SPY (reference), glucose- or xylose-supplemented SPY at 55 °C for 6 h before RNA was extracted. **c** The quality of the extracted RNA was evaluated by bioanalyzer measurements. The most intense RNA bands correspond to the intact 25S and 18S rRNA of the reference, glucose- and xylose-induced cultures. The analysis was performed for three biological replicates of each growth condition

In order to render *C. thermophilum* more amenable to genetic analyses*,* we sought to identify regulatable promoters of gene expression. This might be useful for, among other goals, the regulated expression of (dominant-negative) mutants in this fungus, and hence might facilitate functional studies in vivo. By analogy with mesophilic fungi, we hypothesized that *C. thermophilum* can also regulate the enzymes for its sugar metabolism according to the identity of the sugar provided in the medium. Accordingly, we tested various sugars as the sole source of carbon in minimal media and determined the changes in transcriptome expression. Initially, *C. thermophilum* was cultivated on rich complete cultivation medium (CCM) containing dextrin and saccharose as carbon sources supplemented with tryptone, peptone and yeast extract as complex nutrients (Materials and Methods). In order to identify sugar-regulated genes, we established a minimal medium containing only the salts, peptone and yeast extract (SPY medium) in equal amounts to those provided in the CCM. Addition of 1% (w/v) glucose or xylose then defined the medium as glucose- or xylose-containing, respectively (Materials and Methods). Whereas no mycelium growth was observed on minimal sugar-free medium, fungal colonies grew well on glucose- or xylose-containing media. Owing to the lower nitrogen and carbon content in the minimal media, the radial growth was slower than mycelia grown on the traditional CCM. Interestingly, mycelia grown on glucose- and xylose-containing media had less defined radial growth and lacked the characteristic “segmentation grooves” compared to colonies grown on traditional CCM (Fig. 1a).

### Illumina deep sequencing reveals the transcriptome dynamics depending on the sugar source

Using different glucose- and xylose-supplemented media, we then screened for differentially transcribed genes that are controlled by carbon-regulated promoters. We incubated wildtype mycelia first in liquid minimal medium (SPY) lacking a source of sugar to deplete carbon storage pools in the cells, from which extracted total RNA served as a reference transcriptome. After 48 h, cultures were shifted to a medium containing either glucose or xylose and incubated for 6 h, before total RNA was isolated (Fig. 1b, c). In order to determine the transcriptomes that resultated from these distinct growth conditions, we applied mRNA-directed lllumina deep sequencing for each condition in triplicate [39]. These triplicates showed a high overall consistency, but were significantly different across the three experimental conditions (Suppl. Figure 1). The transcriptomic reads had a length of 50 bp and comprised 75 million reads from the SPY medium, and about 70 million reads from the glucose- and xylose-induced conditions. From each sequencing run, we could map approximately 90% of the obtained sequencing reads onto the reference genome [8, 10] accessible at https://c-thermophilum.bork.embl.de [40] (Table 1).

**Table 1:** Illumina deep sequencing read output

Condition	Biological replicates	Average number of reads (millions)	Percentage of mapped reads	Mapped reads (millions)
Reference	3	75	91.5	69
Glucose	3	68	92.1	62
Xylose	3	70	91.7	64

**Fig. 2:**
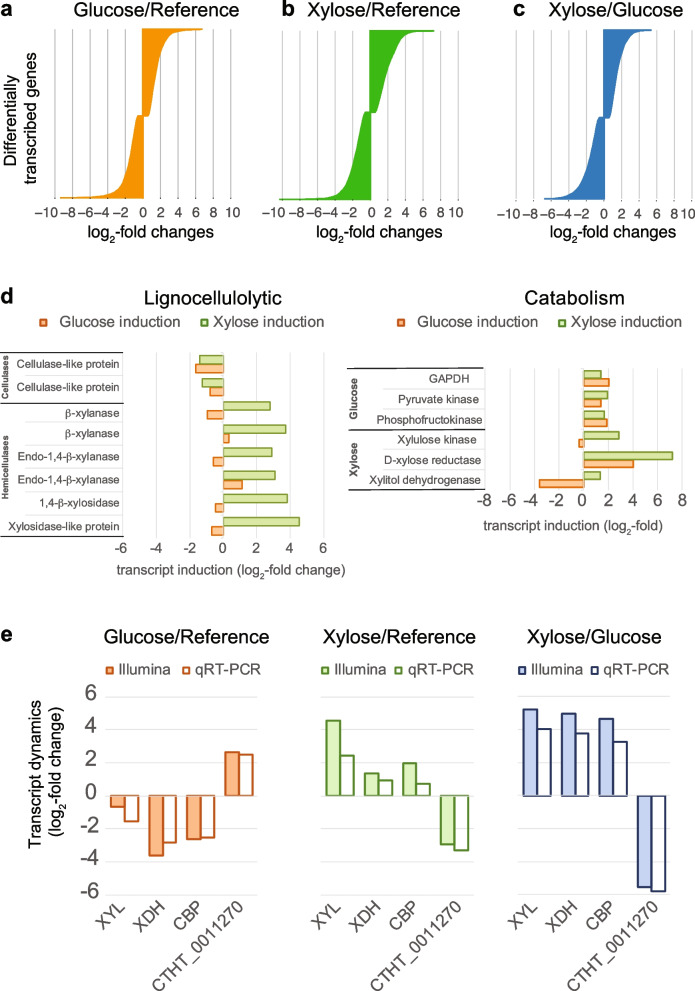
Investigation of sugar-induced transcriptome dynamics in *C. thermophilum*. **a**–**c** The differentially expressed genes (DEGs) after exposure to either glucose or xylose for 6 h are shown. The charts show the significant DEGs as log_2_-fold changes according to Illumina deep sequencing of mRNAs (*p*-adjusted < 0.05). Significant changes in transcripts after glucose induction (3736) compared to the reference condition (**a**), after xylose induction (4685) compared to the reference condition (**b**), and comparing xylose with glucose induction (3750) (**c**). The plotted DEGs are listed in Suppl. Data 1. **d** Selected genes involved in lignocellulolytic and catabolic function were analyzed with respect to their transcriptional dynamics. Shown are the changes in transcripts as log_2_-fold changes after D-glucose (orange) and D-xylose (green) induction in comparison to carbon-deficient reference medium (SPY). The respective gene IDs are given in Suppl. Data 3. **e** The transcriptome dynamics of selected genes were validated by qRT-PCR. The measured changes in transcripts according to Illumina sequencing (filled bars) are shown alongside changes in transcripts according to qRT-PCR (white bars). Transcript dynamics are compared for a β-xylosidase-like gene (XYL), xylitol-dehydrogenase (XDH), a cellulose-binding protein (CBP) and the gene having the strictest activation in glucose compared to in xylose (CTHT_0011270)

Next, we analyzed the differences in transcriptomes between the various growth conditions, for which we could map about 62–69 million 50-bp reads from each condition, allowing us to determine the sugar-dependent dynamics of transcription. First, we sorted the differentially expressed genes (DEGs) according to transcript enrichment upon glucose and xylose induction in comparison to the reference medium (Fig. 2a, b). A comparison of the glucose- and xylose-induced transcriptomes showed that 4258 genes from the approximately 7200 transcripts (~ 60%) were significantly differentially expressed (p-adjusted < 0.05) (Fig. 2c). The DEG plots are shown in Suppl. Data 1.

In order to identify the genes most strictly regulated by sugars in *C. thermophilum*, we identified the 50 most differentially transcribed genes upon glucose and xylose induction (Suppl. Data 2). Of those, 17 genes (34%) were specifically activated by xylose and the other 66% by glucose. Using the Gene Ontology tags from Ref. [39], we found the exclusively glucose-activated genes to be involved in a broad range of cellular processes, ranging from signaling and regulation of transcription to respiratory function, among others. By contrast, the xylose-induced genes are typically involved in xylose metabolism and also translation (Suppl. Figure 2). The three most xylose-induced genes code for enzymes with functions in hemicellulose hydrolysis or xylose catabolism: a xylosidase-like gene (CTHT_0011440), a xylitol dehydrogenase (CTHT_0073860), and a 1,4-β-xylosidase (CTHT_0064690), shown in Fig. 2d. Accordingly, we further examined the transcriptional dynamics of cellulases and hemicellulases from the total transcriptomic dataset (Fig. 2d and Suppl. Data 3). The cellulases did not show significant transcriptional dynamics that depended on exposure to glucose or xylose. By contrast, the hemicellulases, including xylanases and xylosidases, were transcriptionally upregulated in xylose-containing medium compared to the slight induction or even repression in glucose-containing medium. In addition to these lignocellulolytic genes, we examined genes involved in glucose and xylose catabolism. Genes specifically required for the utilization of xylose were either strongly activated in the xylose-containing medium, for example, the genes encoding xylulose kinase (CTHT_0022560) and D-xylose reductase (CTHT_0056950), or strongly repressed in the glucose-containing medium, for example, the xylitol dehydrogenase gene (CTHT_0073860). By contrast, representative genes involved in glycolysis, for example, glyceraldehyde 3-phosphate dehydrogenase (CTHT_0004880), were not differentially transcribed in the presence of either sugar. Thus, *C. thermophilum* constitutively expressed genes needed to live on glucose, whereas the processes needed to mobilize and catabolize xylose were significantly activated in the presence of xylose.

To independently confirm the observed changes in the transcript levels based on our transcriptome-wide analysis, we performed qRT-PCR using TaqMan probes for selected genes involved in xylose metabolism. These genes included a putative cellulose-binding protein (CTHT_0003950), a β-1,4-xylosidase (CTHT_0011440), and the xylitol dehydrogenase gene (CTHT_0073860) next to a noncoding transcript (CTHT_0011270), which was induced most strongly in the glucose conditions. For these four targets, the qRT-PCR analysis revealed highly similar transcriptomic dynamics, as determined by Illumina deep sequencing (Fig. 2e and Suppl. Data). Thus, we concluded that promoters from these genes were bona fide candidates for establishing a sugar-inducible gene expression system.

### Characterization of sugar-regulated promoters in YFP-reporter strains

**Fig. 3:**
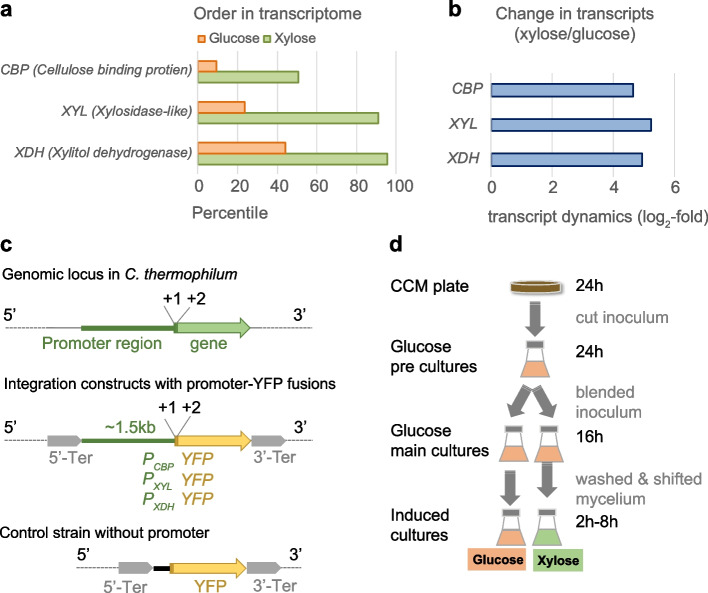
Experimental characterization of xylose inducible promoters in YFP-reporter strains. **a** Relative ranking of transcript abundancies of selected genes in the genome when mycelia were grown in glucose- (orange) or xylose- (green) containing medium. **b** Absolute transcript dynamics as log_2_-fold changes comparing selected genes after the fungal mycelium was incubated in glucose or xylose supplemented media. **c** Architecture of YFP-reporter strains to test xylose-inducible promoter sequences. Promoter regions (shown in green) of the cellulose binding protein (*P*_*CBP*_), β-1,4-xylosidase-like gene (*P*_*XYL*_) and xylitol-dehydrogenase (*P*_*XDH*_), respectively, were cloned as transcriptional fusions upstream of a thermostable YFP reporter gene depicted in yellow. The promotors and the reporter gene, were flanked up- and downstream by a transcription terminator sequence (grey). A control strain was generated, which carried a short oligonucleotide spacer upstream of the YFP gene instead of a promoter region. **d** Experimental workflow of the induction assay. Reporter strains were cultivated in glucose-containing medium to build biomass under repressive conditions, washed and subsequently grown for further 8 h in either fresh glucose-containing medium (glucose) or xylose-containing medium (xylose). Immunoblotting and fluorescence microscopy was performed to monitor YFP expression under the control of the various promoters

Having identified sugar-regulated genes in the genome of *C. thermophilum*, we tested various 5’ regions upstream of those genes for generating a regulatable gene expression system. In order to identify a strong and tightly controlling promoter, we selected promoters from genes with various transcription strengths according to the measured transcripts (Fig. 3a, b). As a method for measuring promoter activities, we sought to connect these promoters to a gene coding for a fast-folding thermostable YFP (ffts-YFP). Importantly, this YFP gene was codon-optimized and the thermostability of the encoded protein was increased by introducing the mutations reported previously [41]. With this reporter system, it was possible to quantify gene expression levels by immunoblotting using an α-GFP antibody, and by measuring fluorescence intensities in the YFP channel using fluorescence microscopy. To clone these promoters, we retrieved the 1.5 kbp DNA fragments spanning the 5’ upstream regions of three xylose-regulated genes in which we expected the regulated promoter region to reside. Those genes encode a cellulose-binding protein (*CBP*) and xylitol dehydrogenase (*XDH*), and the third was a β-1,4-xylosidase-like gene (*XYL*). Accordingly, we denoted the promotor region *P*_*CBP*_, *P*_*XDH*_ and *P*_*XYL*_, respectively. Because the stability of a protein is reported to depend on the most N-terminal residue [42, 43], in the YFP-reporter construct we also included the first two translated codons of the native gene in the respective promoter-carrying region (ATG + NNN) (Fig. 3c). Furthermore, we flanked the promoter-YFP fusions with upstream and downstream terminators to block potential transcriptional background activity from the genomic locus into which the reporter plasmids randomly integrate upon transformation (see the Materials and Methods). The three promoter-carrying reporter constructs were transformed into *C. thermophilum* protoplasts and several independent terbinafine-resistant colonies were selected. To verify YFP expression, the individual transformants were grown on inductive xylose medium and those clones showing strong YFP signals in the immunoblot and fluorescent mycelia during live-cell imaging were selected for further analysis. As a mock control, we generated a *C. thermophilum* strain in which a short DNA spacer sequence was inserted upstream of the YFP gene instead of the cloned promoter region. Owing to the lack of detectable expression, the genotype of this control strain was validated by PCR analysis on gDNA extracted from the transformants (Suppl. Figure 3, 6).

**Fig. 4:**
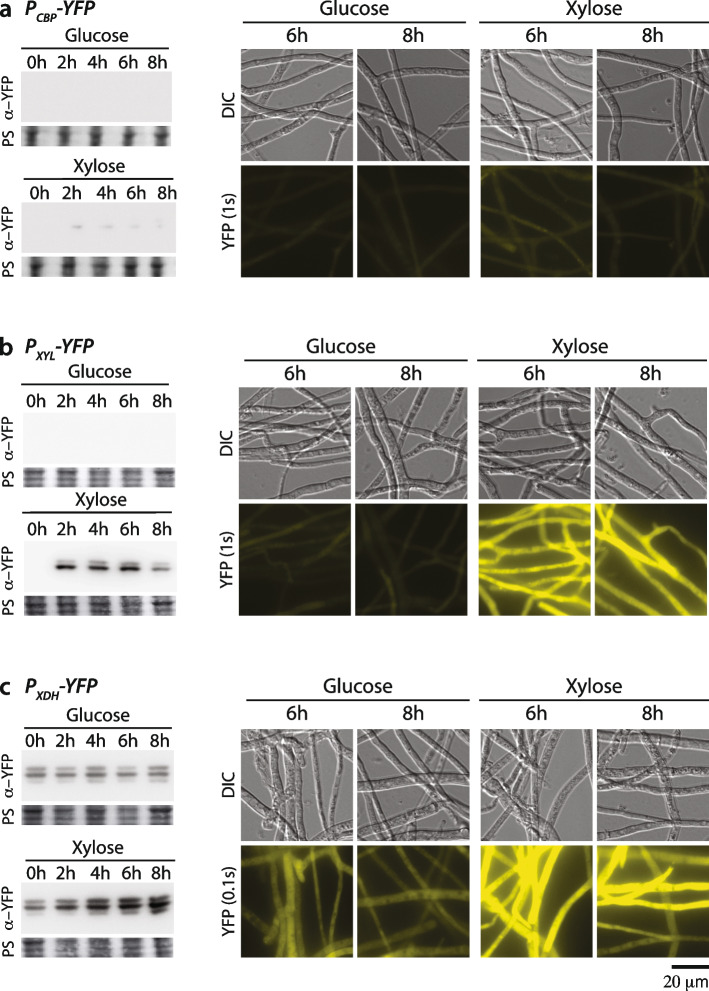
Characterization of carbon-regulated promoters in YFP-reporter strains. Promoter regions from selected glucose-repressed and xylose-activated genes were characterized in YFP-reporter strains. The promoter-YFP-expressing reporter strains were subjected to the induction assay shown in Fig. 2. In essence, reporter strains were grown in glucose-containing medium for 16 h and subsequently shifted to glucose-containing or xylose-containing medium for up to 8 h. Samples were taken before medium exchange (0 h) and at the indicated time points after medium exchange for fluorescence microscopy and immunoblotting against the YFP. YFP expression is shown for P_*CBP*_ (**a**), P_*XYL*_ (**b**) and P_*XDH*_ (**c**). Because no antibody is available for normalization purposes, we loaded equal amounts of protein, as shown by ponceau S (PS) staining. Note that lysates from cultures grown in glucose and xylose were analysed on the same membrane. Exposure times for the Western blot had to be adjusted according to the level of expression of the respective promoter, as follows: P_*CBP*_ (5 min), P_*XYL*_ (1 s)_,_ P_*XDH*_ (10 s). Exposure times were identical for Glu- and Xyl-derived samples. Unprocessed blots/gels are presented in Supplementary Fig. 7

To verify the regulative function of the 5´ promoter region, we evaluated the reporter strains in an induction assay in which we could monitor promoter tightness in repressive glucose-containing medium and induction strength in xylose-containing medium (Fig. 3d). The expressed YFP was measured by immunoblotting before xylose induction and in a time series up to 8 h after induction. Furthermore, protein expression was monitored in vivo by fluorescence microscopy for 6 and 8 h upon induction (Fig. 4). The control strain lacking a promoter upstream of the YFP gene showed no detectable YFP by either immunoblotting or fluorescence microscopy. The *CBP* promoter (P_*CBP*_)-carrying reporter strain, which was predicted to show the weakest induction activity of our selected promoters, did not show any YFP expression in the presence of glucose. By contrast, exposure to xylose-containing medium resulted in a weak but significant YFP immunoblot signal, even at the first time point (2 h induction). However, *in vivo*, a YFP fluorescence signal was barely detectable under glucose- and xylose-grown conditions (Fig. 4a and Suppl. Figure 7). A significantly improved induction rate was observed for the *XYL* promotor (P_*XYL*_). Using exposure times fivefold shorter than that given to the P_*CBP*_-carrying reporter strain, we observed no signal in the immunoblot analysis in samples that originated from glucose-grown cells, but a strongly elevated signal after only 2 h of xylose induction (Fig. 4b). These observations were in agreement with the YFP fluorescence detected, if a YFP exposure time identical to that used for the *P*_*CBP*_ strain was applied. Only an extremely low signal was detected in cells grown in the glucose-containing medium, whereas the xylose-induced cells showed abundant YFP expression under the control of *P*_*XYL*_. This stringent regulation on the protein level was also supported by affinity purification of the YFP by GFP-Trap: a significant yield of pure YFP was detected on Coomassie-stained SDS-PAGE gels exclusively after xylose induction (Suppl. Figure 4, 9). Hence, we concluded that P_*XYL*_ is effectively repressed in the presence of glucose and specifically induced in a xylose-containing medium, allowing the regulated expression of proteins of interest. Finally, we analyzed the *XDH* promoter (P_*XDH*_) in our reporter system; this was the strongest promoter in vivo according to our transcriptome analysis (Fig. 3a). In order to assess expression under the control of P_*XDH*_, we had to further shorten the exposure times to detect a sufficiently strong YFP signal by immunoblotting. For consistency, we cut the exposure time in fluorescence microscopy by about 90% compared to the *P*_*CBP*_ and P_*XYL*_ activity measurements. By using such short exposure times, a moderate YFP signal was detected by immunoblotting and fluorescence microscopy in samples that were harvested from cultures grown in glucose. The detected YFP was then significantly enriched once mycelia were incubated in xylose supplemented medium for 2 h and longer (Fig. 4c and Suppl. Figure S7). In order to compare the protein expression levels promoted by P_*XDH*_ and P_*XYL*_, we sequentially diluted protein samples of the P_*XDH*_ strain and analyzed them by immunoblotting alongside samples isolated from the P_*XYL*_ strain (Suppl. Figure 5 and Suppl. Figure 10). The result indicated that P_*XDH*_ samples that were diluted by a factor of 8 had a similar signal intensity than undiluted P_*XYL*_ samples. Consistent with that, we obtained a similar fluorescence signal for *P*_*XDH*_-YFP-carrying cells with a tenfold shorter induction time used for the *in vivo* microscopy. Since we detected YFP exclusively for the various promoter-carrying reporter strains but not in the control strain, we concluded that the amount of YFP can be correlated with the respective promoter activities. Taken together, our molecular analysis revealed that P_*XYL*_ is highly suitable for stringently controlling the expression of proteins, whereas P_*XDH*_ might be preferred for stronger overexpressions.

### The P_XDH_ regulates a dominant-negative mutant mapping into ribosome biogenesis

**Fig. 5:**
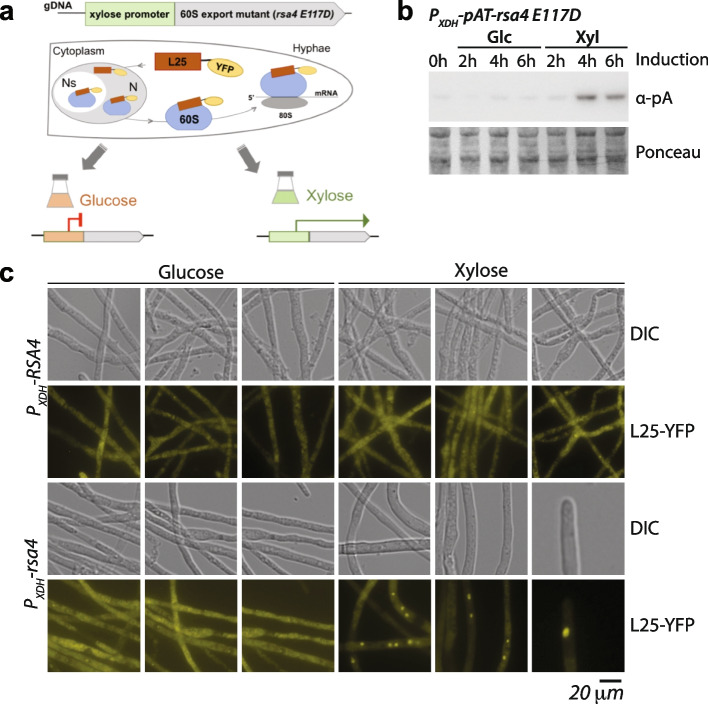
The xylose-inducible P_XDH_ promoter controls a dominant-negative mutant eliciting a 60S export defect. **a** Cartoon showing the reporter assay for monitoring an inducible localization defect of the large 60S subunit using L25-YFP. We constitutively expressed L25-YFP using the hph1 selection marker and introduced additional plasmids that allow overexpressing of the ribosome biogenesis factor pA-Rsa4 (P_XDH_-RSA4) or the mutated protein pA-rsa4 E117D (P_XDH_-rsa4) under the control of P_*XDH*_, using the erg1 selection marker. Based on the rsa4 E117D mutant from yeast, a dominant-negative growth phenotype and the accumulation of L25-YFP in the nucleoplasm is expected upon induction of the mutant protein. **b** Immunoblotting shows the controlled expression of pA-rsa4 E117D on xylose-containing medium at the indicated time points. The uncropped blot is shown in Related file 3. **c** L25-YFP localization in RSA4- and rsa4 E117D-expressing strains under the control of P_*XDH*_. L25-YFP strains expressing pA-RSA4 (*RSA4*) or pA-RSA4 E117D (*rsa4*) under the control of *P*_*XDH*_ were grown for 16 h in glucose-containing medium and then shifted to fresh glucose- or xylose-containing medium for another 4 h. Images were acquired in the DIC and YFP channels. Unprocessed blots/gels are presented in Supplementary Fig. 8

In order to test whether our xylose-inducible promoters are suitable genetic tools for characterizing protein function *in vivo* within *C. thermophilum*, we sought to express a dominant-negative mutant in a regulated manner. Thus, we chose the ribosome biogenesis factor Rsa4, for which a dominant lethal mutant (*rsa4 E114D*) has been described in *S. cerevisiae* [44]. Moreover, structural characterization has shown *sc*Rsa4 to be highly homologous to *ct*Rsa4 [13]. Regulated expression of the *rsa4 E114D* mutant under the control of *P*_*GAL1*_ in yeast elicits a strong growth defect and 60S subunit export arrest in the nucleoplasm upon induction in galactose medium [45]. By contrast, yeast cells grow without restriction in a repressing glucose-based medium. In order to challenge our strongly xylose-inducible *P*_*XDH*_, we created a plasmid coding for a *ctrsa*4 E117D mutant under the control of *P*_*XDH*_ and followed protein induction in the strongest-expressing transformant. As a control, we generated the analogous strain with a *ctRSA4* wildtype allele. To monitor such a dominant-negative 60S export defect as described in yeast studies [45, 46], we created an analogous *C. thermophilum* reporter strain expressing the ribosomal protein Rpl25 fused to ffts-YFP (Fig. 5a). Rpl25-YFP was constitutively expressed under control of the 1.5-kb-spanning 5’ region of *RPL25* and transformed into the respective Rsa4 recipient *C. thermophilum* strains expressing the *ctRSA4* wildtype or the *ctrsa4 E117D* mutant under the control of the *P*_*XDH*_ promotor (Table 2). The resulting strains were subsequently analyzed after inducing for 4 h, the same time after which Rsa4 variants under the control of *P*_*XDH*_ showed strong expression, as demonstrated for pA-Rsa4 E117D (Fig. 5b and Related file 3). Live-cell imaging using the wildtype *RSA4* allele or the *rsa4 E117D* mutant in repressive (glucose containing) medium revealed the subcellular localization of Rpl25-YFP to be the nucleolus and the cytoplasm. By contrast, upon induction of *rsa4 E117D* in the presence of xylose for 4 h, Rpl25-YFP accumulated in the nucleoplasm. This is where Rpl25 joins the maturing 60S subunit, which cannot be efficiently exported into the cytosol (Fig. 5c) caused by the expression of the Rsa4 E117D mutant.

**Table 2:** Strains used in this study

Name	Genotype	Purpose
*P*_*CBP*_*-YFP*	*P*_*CBP*_*-YFP, erg1*	YFP expression under control of *P*_*CBP*_
*P*_*XYL*_*-YFP*	*P*_*XYL*_*-YFP, erg1*	YFP expression under control of *P*_*XYL*_
*P*_*XDH*_*-YFP*	*P*_*XDH*_*-YFP, erg1*	YFP expression under control of *P*_*XDH*_
*Control*	*Linker-YFP, erg1*	Testing specificity of the reporter construct
*P*_*XDH*_*-pA-RSA4*	*P*_*XDH*_*-pA-RSA4. erg1*	Rsa4 wt expression under control of *P*_*XDH*_
*P*_*XDH*_*-pA-rsa4 E117D*	*P*_*XDH*_*-pA-rsa4 E117D, erg1*	Rsa4 E117D expression under control of *P*_*XDH*_
*P*_*XDH*_*-pA-RSA4*	*P*_*RPL25*_*-RPL25-YFP, hph1* + *P*_*XDH*_*-pA-RSA4. erg1*	RpL25-YFP localization in inducible wildtype Rsa4 background
*P*_*XDH*_*-pA-rsa4 E117D*	*P*_*RPL25*_*-RPL25-YFP, hph1* + *P*_*XDH*_*-pA-rsa4 E117D, erg1*	RpL25-YFP localization in inducible mutant Rsa4 E117D background

Thus, we obtained the first evidence that P_*XDH*_ is suitable for regulating the expression of a dominant-negative mutant. Future studies might further exploit the promoters presented here as powerful genetic tools for manipulating thermophiles for research or industrial purposes.

## Discussion

In this study, we identify and characterize endogenous sugar-regulated promoters for *C. thermophilum*, as regulated protein expression is a versatile tool in model organisms. However, inducible promoter systems have not yet been established for this thermophilic eukaryote, even though it continues to be exploited for fundamental research and industrial applications [23, 42, 43]. Owing to its tolerance to high temperatures—growth being optimal at around 50 °C—it is somewhat unsuited to applying heterologous regulatable promoters that are commonly utilized in related mesophilic organisms [32–34]. Either proteins (e.g. transcription factors) have to be adapted to increase their thermostability, as was the case for our thermostable YFP reporter, or workflows have to be modified (e.g. the stable expression of heterologous resistance markers such as hygromycin B is only possible at lower growth temperatures) [15]. To circumvent this problem, we set out to identify endogenous promoters within the *C. thermophilum* genome by conducting an extensive transcriptomic analysis. A further advantage of using the carbon source to induce regulated gene expression is that it is an inexpensive and nonhazardous strategy for regulating protein expression in *C. thermophilum*.

Our transcriptomic analysis revealed that a multitude of genes involved in xylose metabolism are upregulated in *C. thermophilum* cultured in medium containing xylose as the sole carbon source. This result is in good agreement with our expectations, as these genes are known to be required for carbon and energy acquisition [44, 47]. Subsequent molecular characterization of the DEGs indicated that *P*_*XYL*_ and *P*_*XDH*_ are the most strictly regulated promoters that adopt glucose or xylose as repressing and/or inducing molecules. The major difference between the two appeared to be the approximately tenfold higher basal activity of the *P*_*XDH*_*.* Thus, *P*_*XDH*_ is beneficial if a high expression level is required and a certain level of leakiness is tolerable. For other experiments in which small amounts of expressed proteins are investigated [48], the *P*_*XYL*_ could serve as a strictly glucose-repressed and xylose-activated promoter. However, at this level of characterization we cannot rule out that the protein expression is also (partially) influenced by post-transcriptional or translational effects. The xylose-activatable promoters investigated here, are coupled to quick activation responses, reflected in the protein levels within the first two hours after induction. This remarkably fast onset of protein expression lessens the risk of undesired pleiotropic effects due to prolonged expression of mutant genes. As a proof of principle, we controlled the expression of the dominant-negative ribosome biogenesis mutant *rsa4 E117D* in *C. thermophilum*. Here, we chose to control the mutated allele with the *XDH* promotor, as ribosome biogenesis factors in general show overall high levels of expression, as our transcriptomic data set confirmed. Indeed, the expression of Rsa4 E117D was subject to a significant 60S ribosome export defect, whereas no defect was detected under repressed conditions. In our setup, the transformation cassette is randomly integrated into the genome of *C. thermophilum*. Thus, the number of integrations and the genomic locus of integration might influence the measured expression level of the target genes. However, to better control this issue, future studies could use a recently described strain of *C. thermophilum* that has been genetically modified to enable site-specific integration of DNA by homologous recombination [49]. Using homologous recombination-directed in-locus integration of the conditional promoters identified here would eliminate the aforementioned issues concerning potential genetic variance within transformants. Importantly, combining both methods would allow the function of uncharacterized or essential genes to be investigated. Furthermore, the replacement of an endogenous promoter by a sugar-regulated promoter might enable the phenotype resulting from these genes to be studied under depleted conditions, as was successfully performed in *N. crassa* using the copper-inducible promoter *P*_*tcu*_-1 [31].

We expect that our molecular characterization of xylose-regulated promotors will stimulate further enhancements of regulatable protein expression in *C. thermophilum*. By contributing the first regulatable promoters to the study of this thermophilic fungus, we have expanded the genetic tool set that equips us to further exploit the valued properties of *C. thermophilum.*

## Materials and methods

### Strain generation

The wildtype strain *Chaetomium thermophilum var. thermophilum* [9] DSMZ 1495 was used. Genetically modified *C. thermophilum* strains were generated by random integration of DNA constructs as previously described [15, 25]. The strains used in this study are listed in Table 2.

### Cultivation media

The traditionally used CCM, consisting of 0.5 g NaCl, 0.65 g K_2_HPO_4_∙3H_2_O, 0.5 g MgSO_4_∙7H_2_O, 0.01 g Fe(III) SO_4_ hydrate, 5 g tryptone, 1 g peptone, 1 g yeast extract, 3 g sucrose, and 15 g dextrin (potato) per liter (pH 7.0), was used to recover *C. thermophilum* strains from preserved spores. A minimal medium, that is, the CCM with the nutrients sucrose, dextrin and tryptone omitted, was also prepared, which we termed SPY since it contained only the salts, peptone and yeast extract in the same quantities per liter (pH 7.0) as the CCM. For solid media, agar (20 g per liter) was added. Glucose- and xylose-supplemented media contained 1% (w/v) D-glucose and D-xylose, respectively.

### DNA procedures

Plasmids were generated by restriction cloning, as described by Sambrook et al. [50]. *E. coli* DH5α was used for plasmid amplification using standard procedures. Genomic DNA from *C. thermophilum* was isolated by phenol–chloroform extraction according to Al-Samarrai and Schmid [51]. The sequences of oligonucleotides used for PCR reactions are listed in Table 3.

**Table 3:** Oligonucleotides used in this study

Name	Oligonucleotide (5’–3’)	Purpose
NdeI-YFP-fw	GCCCATATGCGCAAGGGCGAGGAGCTTTTC	Thermostable YFP
FseI-YFP-rv	TCTGGCCGGCCTTAAACGCTGGCAGCGTAGTTCTC	
Ter-4700-XbaI-f	GCCTCTAGATAAGGAAGATGAAGAGAAGCAAGAGG	5’ terminator
Ter-4700-MCS-r	GAATTCGGGAAGGCGCGCCAAAACGCGTAGGTATATTAACTTACTCGATACTACTTC	
Pct_0003950-fw	GCCGAATTCCATAGACTTATAAAACAGTCTGCCCAC	P_CBP_
Pct_0003950-rv	GCCCATATGCTTCATCGTAATCTTTTTCGCTTACAC	
Pct_0011440-fw	GCCGAATTCCTCAGCTTTTCTTGGCAGCTAGATG	P_XYL_
NotI-FseI-YFP-rv	GCGGCCGCAAGGGCCGGCCTTAAACGCTGGCAGCGTAGTTCTC	
Pct_0073860-fw	GCCGAATTCCAACAACCGCAATCTGCCGCAA	*P*_*XDH*_
Pct_0073860-fw	CAACAACCGCAATCTGCCGCAA	
no_promoter_fw	AATTCCCTTCATCTGTCTAGAACAACGTGCA	Control linker
no_promoter_rv	TATGCACGTTGTTCTAGACAGATGAAGGG	
EcoRI-pAT-fw	GCCGAATTCATGGCAGGCCTTGCGCAACAC	pAT-tag
Nde-pAT-rv	GCCCATATGATGAGCACCACCTTGAAAATATAAATTTTCAG	
NdeI-RSA4-f	GCCCATATGGCTACATTAGCCCCTCCACC	RSA4/rsa4 E117D
NotI-RSA4-Stop-r	GCCGCGGCCGCCTATTAGTTTCTCCATGTCCGAACAGCC	
5'UR_RpL25-EcoRI-fw	GCCGAATTCTCAGAAACATTAAGTATGTTATGAACCGG	*P*_*RPL25*_*-RPL25-YFP*
L25-linker-nostop-NdeI-rv	GCCCATATGTGATCCGACCAGGCCGAGCTTGGTGG	

The generated plasmids that were transformed in *C. thermophilum* are listed in Table 4.

**Table 4:** Plasmids used in this study

Plasmid	Genotype	Selection marker
pSR31	pRSF_Ter-P_CBP_-YFP-Ter	*erg1*
pSR32	pRSF_Ter-P_XYL_-YFP-Ter	*erg1*
pSR35	pRSF_Ter-P_XDH_-YFP-Ter	*erg1*
pSR41	pRSF_Ter- linker-YFP-Ter	*erg1*
pSR72	pRSF_Ter-P_RPL25_-RPL25-YFP	*hph1*
pSR99	pBS_P_XDH_-pAT-RSA4	*erg1*
pSR100	pBS_P_XDH_-pAT-RSA4_E117D	*erg1*

### Extraction of total RNA from C. thermophilum

Total RNA was isolated from *C. thermophilum* cultures using the SV Total RNA extraction kit (Promega) according to the manufacturer's instructions. Mycelium was grown for 20 h on a CCM plate, harvested, cut into small pieces, and transferred to 100 ml of glucose-containing medium in a 250 ml baffled flask. The cultures were incubated at 50 °C at 100 rpm for 18 h and subsequently homogenized at 6000 rpm for 2 min using a GM200 blender (Retsch, Haan, Germany). Two milliliters of homogenized *C. thermophilum* cultures were transferred to carbon-deficient SPY medium and grown for another 42 h. Then, RNA was extracted in triplicate from mycelium that was propagated in SPY medium and 6 h after subsequent growth in glucose- and xylose-containing media. In brief, cultures were harvested by sieving, thoroughly washed with 500 ml water (approx. 50 °C) and ground to a fine powder in a liquid-nitrogen-cooled mortar. Approximately 100 mg of cell powder was resuspended in 600 μl of lysis buffer, to which 1200 μl of dilution buffer was added. Samples were further processed according to the manufacturer's instructions.

### Induction assay for monitoring YFP expression in promoter-YFP-reporter strains

*C. *thermophilum var. *thermophilum* mycelium (DMSZ 1495) was activated from spores and propagated on CCM agarose plates as described previously [25]. Mycelia were grown for 20 h on CCM agar plates, scraped from the plates and cut into small pieces as inoculum for liquid pre-cultures which were then propagated for 24 h in baffled flasks containing 600 ml of glucose medium at 50 °C with agitation at 95 rpm. The cultures were grown overnight then shredded at 6000 rpm for 2 min in a GM200 blender (Retsch, Haan, Germany) and 70 ml of homogenized mycelia was used to grow 2 l of main cultures for 16 h in glucose-containing medium. The mycelia were then strained through a metal sieve (ISO 3310–1, pore size 100 µm), washed thoroughly with hot water (approx. 50 °C), and shifted to 2 l of preheated glucose- or xylose-containing medium for further cultivation for up to 8 h.

### Western blotting

Fifty ml of submerged *C. thermophilum* cultures were collected from liquid cultures, harvested with a metal sieve, dried, and immediately frozen at − 20 °C until the whole cell lysates were prepared. Frozen mycelium was resuspended in 800 µl NB-HEPES lysis buffer [20 mM HEPES, pH 7.5, 150 mM NaCl, 50 mM KOAc, 2 mM Mg(OAc)_2_, 1 mM dithiothreitol, 5% glycerol (v/v), 0.1% NP40 (v/v), 40 µl/ml SIGMAFAST protease inhibitor cocktail (Sigma–Aldrich)], and mechanically lysed with 500 µl of zirconia beads (0.5 mm diameter, Carl Roth, Karlsruhe, Germany) in a Precellys 24 homogenizer (Bertin Instruments, Montigny-le-Bretonneux, France) in four runs at 5,000 rpm, 2 × 20 s at 4 °C. Lysates were cleared by centrifugation at 14,000 rpm at 4 °C for 25 min. The protein concentration was determined using *A*_280_ measurements with a NanoDrop 2000 spectrophotometer (Thermo Scientific, Waltham, MA), and normalized to equal total protein concentrations of 10 µg/µl in SDS loading buffer. Ponceau S solution was used to check for equal protein transfer on the membrane (Serva, Heidelberg, Germany). For anti-YFP-directed immunoblotting, a mouse anti-GFP antibody was used (1:3000, 1,181,446,001, Roche) followed by HRP-conjugated goat anti-mouse-IgG antibodies (1:3000, 170–6516, BioRad). For immunoblotting of pA-tagged proteins, anti-pA-HRP-conjugated antibodies (1:3000, P1291, Merck) were used. To image the expressed YFP, an ImageQuant LAS 4000 mini biomolecular imager (GE Healthcare, Chicago, IL) was used.

### Microscopy

To observe YFP expression and localization in YFP-reporter strains, 50 ml of submerged *C. thermophilum* cultures were harvested, washed thoroughly in hot water (50 °C) and resuspended in an equal volume of hot water (50 °C). After homogenization of the culture with a sterile hand blender for 45 s, a sample was applied to an agarose pad (1% agarose in 50 mM Tris–EDTA, pH 8) and inspected with a Zeiss Axio Imager Z1 (Zeiss, Oberkochen, Germany) equipped with a 63 × NA 1.4 Plan-Apochromat oil-immersion objective lens.

### GFP*-*Trap^*©*^

For affinity purification of the YFP protein from *C. thermophilum*, a strain expressing the YFP reporter under the control of the xylose-inducible *P*_*XYL*_ was cultivated in 750 ml of CCM and subjected to the induction assay as described above. The mycelium was harvested 6 h after the medium shift, lysed in a cryogenic cell mill (MM400, Retsch, Germany) and YFP was purified from the cleared supernatants using anti-GFP-coated agarose beads (GFP-Trap; Chromotek, Munich, Germany) according to the manufacturer’s instructions.

### qRT*-*PCR

Extracted total RNA from *C. thermophilum* was used for complementary DNA (cDNA) synthesis. Reverse transcription was performed using the Maxima First Strand cDNA Synthesis Kit for RT-qPCR (Thermo Scientific) following the manufacturer’s instructions. The DNA sequences of the target genes and the *rbp2* normalizer gene were obtained from the *C. thermophilum* Genome Resource [40]. For qRT-PCR, TaqMan probes were designed with help of the Universal ProbeLibrary (UPL) Assay Design Center (Roche). The TaqMan pRT-PCR kit (Thermofisher) was used according to the provider's instruction. All qRT-PCR reactions were measured in three technical and two biological replicates. For relative quantitation of transcript dynamics, the ΔΔCT quantitation method was applied according to Ref. [52]. The oligonucleotides and probes used for qRT-PCR are listed in Table 5.

**Table 5:** Oligonucleotides and probes used for qRT-PCR measurements

Gene	Description	Forward primer (5’–3’)	Reverse primer (5’–3’)	UPL probe
CTHT_0045510	Normalizer	atttgtacggcgaagacgat	cttgatcgtacgggcactc	15
CTHT_0011440	XYL	gaccacgcatcactccatc	cgcagtcgtgatggaaca	81
CTHT_0003950	CBP	gacaactactcgggcaccat	gatgcgcaacaggtagtcac	4
CTHT_0073860	XDH	gcatgggcaaggctgata	gccatagcggaaggaacc	60
CTHT_0011270	Noncoding	cctgctctcagatcgaaaaga	gcttgatctgcaagagtgga	71

### Illumina deep sequencing

Sequencing libraries were prepared using the NEBNext Ultra II Directional RNA Preparation Kit for Illumina with NEBNext PolyA Selection Module, and NEBNext Multiplex Oligos for Illumina. Library preparation and single-end sequencing was performed by the CellNetworks Deep Sequencing Core Facility (Heidelberg, Germany) on an Illumina NextSeq 500 platform.

### Analysis of sequencing data

Quality checking of raw reads was done using FastQC [53]. Reads were mapped to the C. thermophilum genome using Tophat2 [54] using a bowtie index created from the genome from Ensembl release 28. Mapping parameters were set to allow intron sizes between 40 and 5000 (-i 40 -I 5000), based on gap-checks of the corresponding reference genome file (.gtf). Differential expression analysis and visualisation were done using R [55]. The DESeq2 software package was used to perform differential expression analysis, using the three induction types as variables [56]. DESeq2 fits negative binomial generalized linear models for each gene and uses the Wald test for significance testing. The DESeq2 object was created from the mapped samples with summarizeOverlaps from the GenomicAlignments package, using the corresponding reference genome files (.gtf) file as the gene source [55–57]. Bar plots of the significantly changed genes were created using the ggplot2 package [58] and heatmaps of the changes were created using gplots package [59]. Analysis and visualization codes can be requested from G.S.

## Supplementary Information

**Additional file 1.** **Supplementary Figure 1**. Transcriptomic similarity among the various treated samples. The conformities among the three biological replicates that were analyzed by single end Illumina sequencing for the reference (R), the glucose (G) and xylose (X) treated cultures. The heatmap shows the Euclidean distance matrix (from dark blue for zero distance to white for large distance) for the nine sequencing samples after regularized logarithmic transformation. The dendrogram represents a hierarchical clustering.

**Additional file 2.** **Supplementary Figure 2. **Heat map of the 50 most significant changes between glucose- and xylose-treated samples. Each line represents one gene for which row-normalised values from regularized logarithm transformed data are shown in color visualizing weak (bright) and strong (blue) transcribed genes. Pie charts show the functional characterisations of the genes upregulated in each treatment. The individual gene-IDs and transcript dynamics are collected in Supplementary Data 2.

**Additional file 3.** **Supplementary Figure 3.** Genotype verification of the control reporter strain without promoter. The control strain was verified by diagnostic PCR on extracted gDNAs from the obtainedtransformants. The primer pairs (red arrow) were selected as such that theyanneal upstream of the spacer sequence and inside the YFP-gene, producing a 180 bp sized PCR product when the YFP-cassette is present in the genome. The reporter cassette carrying plasmid was used as positive control and gDNA from wildtype mycelia as negative control for the PCR. The representative clone 10 was used further in this study. The uncropped agarose gel is shown in Supplementary Figure 6.

**Additional file 4.** **Supplementary Figure 4. **Characterization of affinity-purified ffts-YFP protein under control of the P_XYL_ promotor. Before induction in xylose medium (0h) and six hours afterwards (6h), the expressed YFP-protein was affinity-purified, using anti-GFP coated agarose beads (GFP-Trap). The purified eluates are shown on a Coomassie stained SDS-gel. The bands were also analyzed by mass spectrometry. Whilst YFP_1 corresponds to the full length YFP protein, the variants YFP_2 and YFP_3 showed to be C-terminally truncated, resulting in accordingly lower molecular weight variants. The uncropped SDS-PAGE is shown in Supplementary Figure 9.

**Additional file 5.** **Supplementary Figure 5.**YFP-Induction strength comparison under control of the P_XDH_ and the P_XYL_. Promoter-YFP carrying reporter strains were grown for 16h in inductive xylose medium and whole cell lysates were subsequently analysed by immuno-blotting using an anti-YFP directed antibody. SDS samples from the P_XDH_ -YFP carrying reporter strain were subjected to a 2-fold dilutions series and blotted next to non-diluted SDS samples from a XYL-YFP carrying reporter strain. Equal amounts of protein inundiluted SDS-samples were loaded, as shown by Ponceau S staining. The 8-fold diluted SDS-samples from the P_XDH_ –YFP strain show similar YFP to undiluted SDS-samples from the P_XDH_ -YFP strain (bold). The uncropped membrane is presented in Supplementary Figure 10.

**Additional file 6.** **Supplementary Figure 6**. unprocessed data related to Supplementary Figure 3.

**Additional file 7.** **Supplementary Figure 7**. unprocessed data related to Figure 4.

**Additional file 8.** **Supplementary Figure 8**. unprocessed data related to Figure 5.

**Additional file 9.** **Supplementary Figure 9**. unprocessed data related to Supplementary Figure 4.

**Additional file 10.** **Supplementary Figure 10**. unprocessed data related to Supplementary Figure 5.

**Additional file 11.** **Supplementary Data 1**. All differentially expressed genesidentified by Illumina deepsequencing.

**Additional file 12.** **Supplementary Data 2**. Gene IDs of the 50 most significant changes between glucose- and xylose treated samples.

**Additional file 13.** **Supplementary Data 3**. Gene IDs of selected genes involved in lignocellulolytic and catabolic functions.

**Additional file 14.** **Supplementary Data 4.** qRT measurements of selected genes in comparison to Illumina sequencing.

## Data Availability

The RNA-seq and genome annotation data are deposited in the Gene Expression Omnibus (GEO) database under the accession number GSE214043 (https://www.ncbi.nlm.nih.gov/geo/query/acc.cgi?acc=GSE214043).
