# Supplementary material for: Identification and characterization of sugar-regulated promoters in Chaetomium thermophilum
Source: BMC Biotechnol. 2023 Jul 8;23:19. doi: 10.1186/s12896-023-00791-9 (PMC10329369; doi:10.1186/s12896-023-00791-9)
Supplement: Supplementary file 1 — Additional file 1. Supplementary Figure 1. Transcriptomic similarity among the various treated samples. The conformities among the three biological replicates that were analyzed by single end Illumina sequencing for the reference (R), the glucose (G) and xylose (X) treated cultures. The heatmap shows the Euclidean distance matrix (from dark blue for zero distance to white for large distance) for the nine sequencing samples after regularized logarithmic transformation. The dendrogram represents a hierarchical clustering. [file 12896_2023_791_MOESM1_ESM.pdf]

Supplementary Figure 1

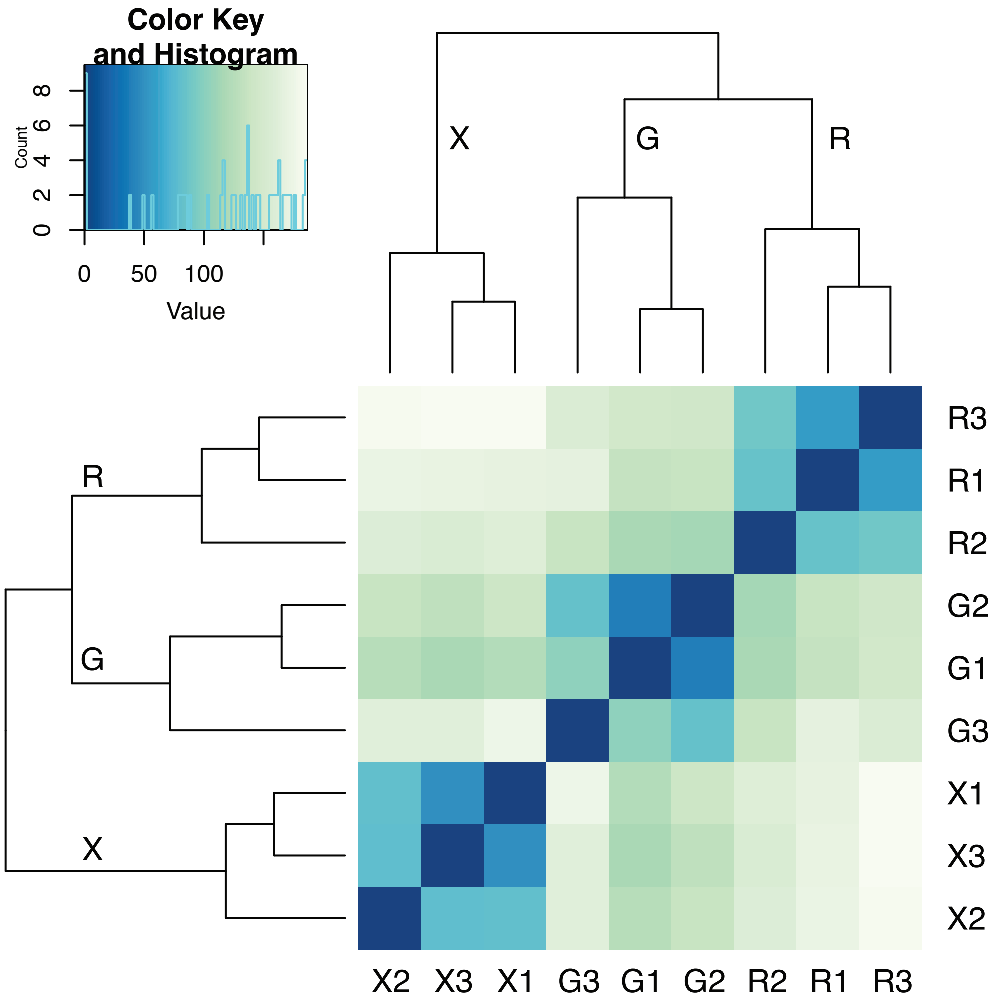

Supplementary Figure 1: Transcriptomic similarity among the various treated samples. The conformities among the three biological replicates that were analyzed by single end Illumina sequencing for the reference (R), the glucose (G) and xylose (X) treated cultures. The heat map shows the Euclidean distance matrix (from dark blue for zero distance to white for large distance) for the nine sequencing samples after regularized logarithmic transformation. The dendrogram represents a hierarchical clustering.
