# Supplementary material for: Identification and characterization of sugar-regulated promoters in Chaetomium thermophilum
Source: BMC Biotechnol. 2023 Jul 8;23:19. doi: 10.1186/s12896-023-00791-9 (PMC10329369; doi:10.1186/s12896-023-00791-9)
Supplement: Supplementary file 2 — Additional file 2. Supplementary Figure 2. Heat map of the 50 most significant changes between glucose- and xylose-treated samples. Each line represents one gene for which row-normalised values from regularized logarithm transformed data are shown in color visualizing weak (bright) and strong (blue) transcribed genes. Pie charts show the functional characterisations of the genes upregulated in each treatment. The individual gene-IDs and transcript dynamics are collected in Supplementary Data 2. [file 12896_2023_791_MOESM2_ESM.pdf]

Supplementary Figure 2

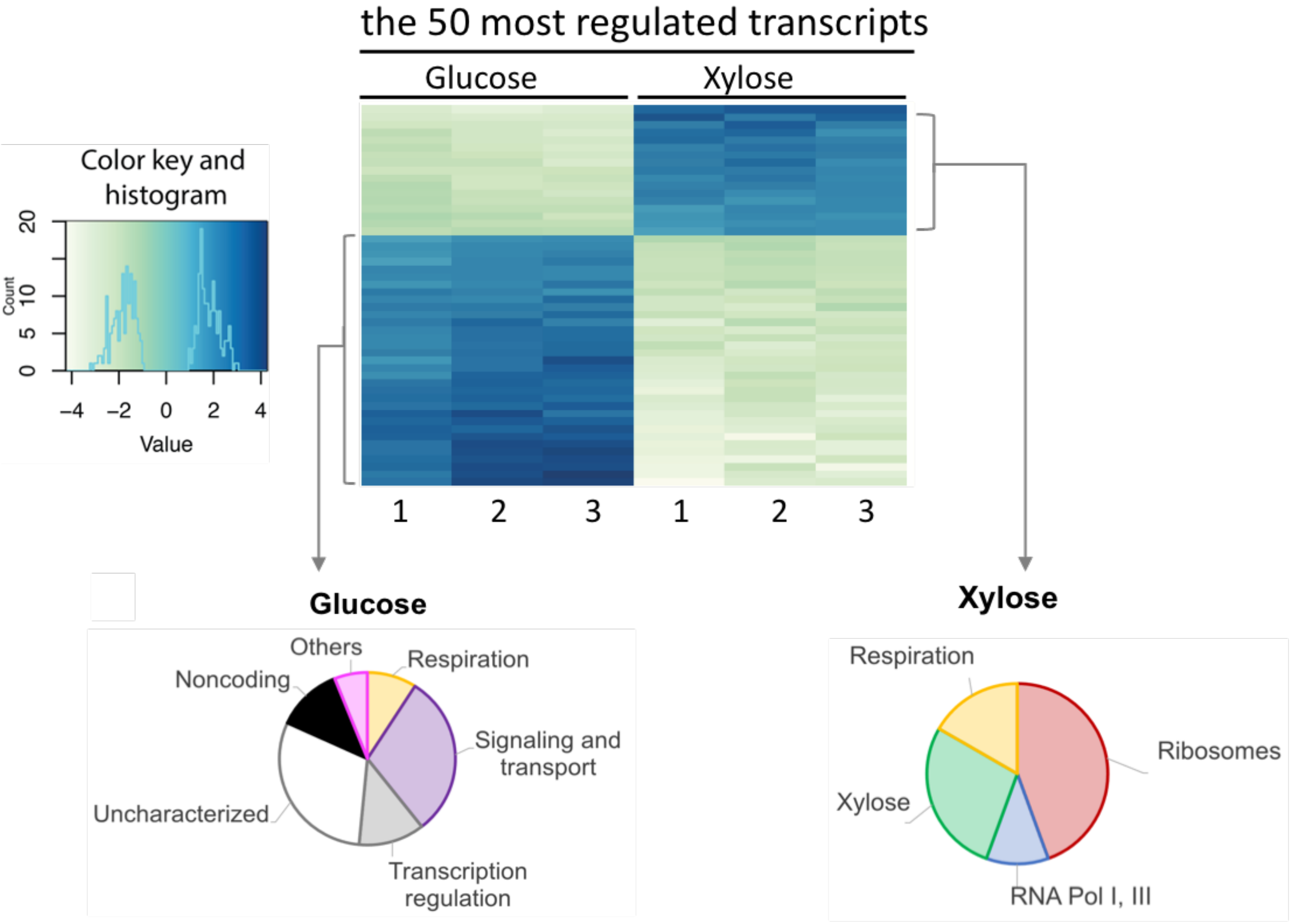

Supplementary Figure 2: Heatmap of the 50 most significant changes between glucose- and xylose-treated samples. Each line represents one gene for which row-normalised values from regularized logarithm transformed data are shown in color visualizing weak (bright) and strong (blue) transcribed genes. Pie charts show the functional characterisations of the genes upregulated in each treatment. The individual gene-IDs and transcript dynamics are collected in Supplementary Data 2.
