# Supplementary material for: Identification and characterization of sugar-regulated promoters in Chaetomium thermophilum
Source: BMC Biotechnol. 2023 Jul 8;23:19. doi: 10.1186/s12896-023-00791-9 (PMC10329369; doi:10.1186/s12896-023-00791-9)
Supplement: Supplementary file 3 — Additional file 3. Supplementary Figure 3. Genotype verification of the control reporter strain without promoter. The control strain was verified by diagnostic PCR on extracted gDNAs from the obtainedtransformants. The primer pairs (red arrow) were selected as such that theyanneal upstream of the spacer sequence and inside the YFP-gene, producing a 180 bp sized PCR product when the YFP-cassette is present in the genome. The reporter cassette carrying plasmid was used as positive control and gDNA from wildtype mycelia as negative control for the PCR. The representative clone 10 was used further in this study. The uncropped agarose gel is shown in Supplementary Figure 6. [file 12896_2023_791_MOESM3_ESM.pdf]

Supplementary Figure 3

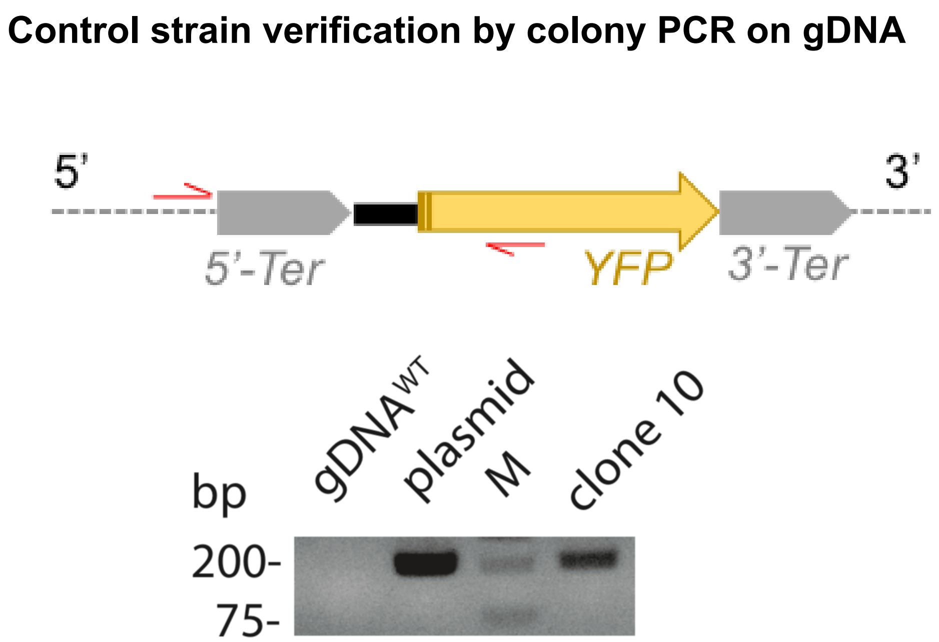

Supplementary Figure 3: Genotype verification of the control reporter strain without promoter. The control strain was verified by diagnostic PCR on extracted gDNAs from the obtained transformants. The primer pairs (red arrows) were selected as such that they anneal upstream of the spacer sequence and inside the YFP-gene, producing a 180 bp sized PCR product when the YFP-cassette is present in the genome. The reporter cassette carrying plasmid was used as positive control and gDNA from wildtype mycelia as negative control for the PCR. The representative clone 10 was used further in this study. The uncropped agarose gel is shown in Supplementary Figure 6.
