# Supplementary material for: Identification and characterization of sugar-regulated promoters in Chaetomium thermophilum
Source: BMC Biotechnol. 2023 Jul 8;23:19. doi: 10.1186/s12896-023-00791-9 (PMC10329369; doi:10.1186/s12896-023-00791-9)
Supplement: Supplementary file 4 — Additional file 4. Supplementary Figure 4. Characterization of affinity-purified ffts-YFP protein under control of the PXYL promotor. Before induction in xylose medium (0h) and six hours afterwards (6h), the expressed YFP-protein was affinity-purified, using anti-GFP coated agarose beads (GFP-Trap). The purified eluates are shown on a Coomassie stained SDS-gel. The bands were also analyzed by mass spectrometry. Whilst YFP_1 corresponds to the full length YFP protein, the variants YFP_2 and YFP_3 showed to be C-terminally truncated, resulting in accordingly lower molecular weight variants. The uncropped SDS-PAGE is shown in Supplementary Figure 9. [file 12896_2023_791_MOESM4_ESM.pdf]

Supplementary Figure 4

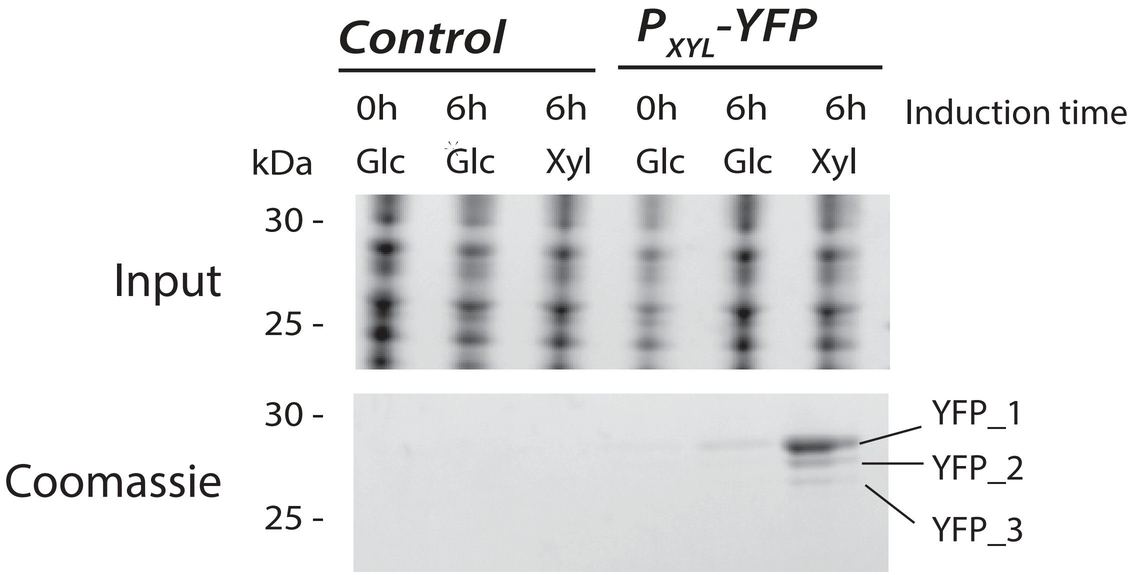


**Supplementary Figure 4:** Characterization of affinity-purified ffts-YFP protein under control of the PXYL. Before induction in xylose medium (0h) and six hours afterwards (6h), the expressed YFP-protein was affinity purified, using anti-GFP coated agarose beads (GFP-Trap©). The purified eluates are shown on a Coomassie stained SDS-gel. The bands were also analyzed by mass spectrometry. Whilst YFP_1 corresponds to the full length YFP protein, the variants YFP_2 and YFP_3 showed to be C-terminally truncated, resulting in accordingly lower molecular weight variants.
The uncropped SDS-PAGE is shown in Supplementary Figure 9.
