# Supplementary material for: Identification and characterization of sugar-regulated promoters in Chaetomium thermophilum
Source: BMC Biotechnol. 2023 Jul 8;23:19. doi: 10.1186/s12896-023-00791-9 (PMC10329369; doi:10.1186/s12896-023-00791-9)
Supplement: Supplementary file 5 — Additional file 5. Supplementary Figure 5.YFP-Induction strength comparison under control of the PXDH and the PXYL. Promoter-YFP carrying reporter strains were grown for 16h in inductive xylose medium and whole cell lysates were subsequently analysed by immuno-blotting using an anti-YFP directed antibody. SDS samples from the PXDH -YFP carrying reporter strain were subjected to a 2-fold dilutions series and blotted next to non-diluted SDS samples from a XYL-YFP carrying reporter strain. Equal amounts of protein inundiluted SDS-samples were loaded, as shown by Ponceau S staining. The 8-fold diluted SDS-samples from the PXDH –YFP strain show similar YFP to undiluted SDS-samples from the PXDH -YFP strain (bold). The uncropped membrane is presented in Supplementary Figure 10. [file 12896_2023_791_MOESM5_ESM.pdf]

Supplementary Figure 5

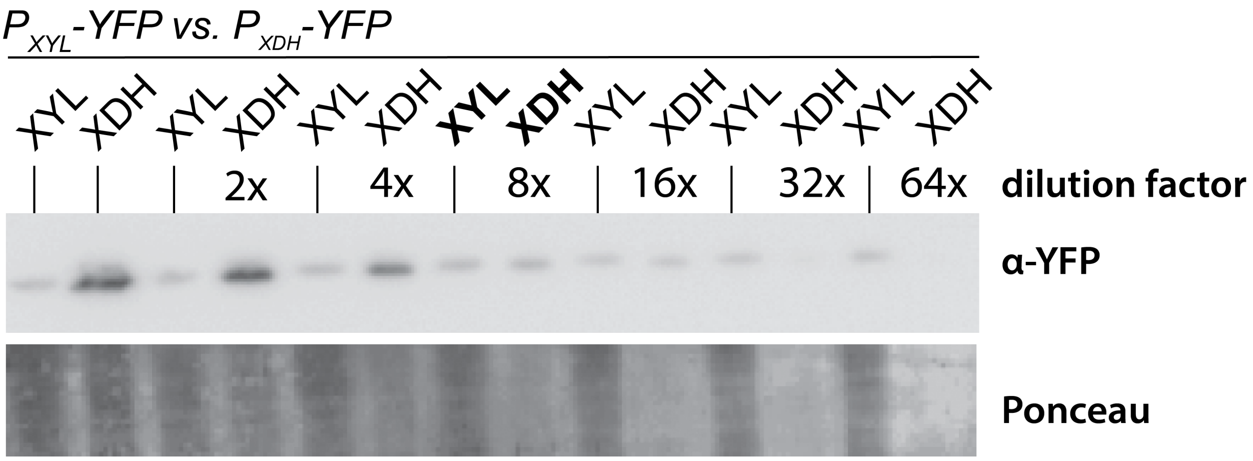

Supplementary Figure 5: YFP-Induction strength comparison under control of the PXDH and the PXYL. Promoter-YFP carrying reporter strains were grown for 16h in inductive xylose medium and whole cell lysates subsequently analysed by immuno-blotting using an anti-YFP directed antibody. SDS samples from the PXDH -YFP carrying reporter strain were subjected to a 2-fold dilutions series and blotted next to non-diluted SDS samples from a XYL-YFP carrying reporter strain. Equal amounts of protein in undiluted SDS-samples were loaded, as shown by Ponceau S staining. The 8-fold diluted SDS-samples from the PXDH -YFP strain show similar YFP to undiluted SDS-samples from the PXDH -YFP strain (bold). The uncropped membrane is presented in Supplementary Figure 10.
