# Supplementary material for: Identification and characterization of sugar-regulated promoters in Chaetomium thermophilum
Source: BMC Biotechnol. 2023 Jul 8;23:19. doi: 10.1186/s12896-023-00791-9 (PMC10329369; doi:10.1186/s12896-023-00791-9)
Supplement: Supplementary file 6 — Additional file 6. Supplementary Figure 6. unprocessed data related to Supplementary Figure 3. [file 12896_2023_791_MOESM6_ESM.pdf]

Supplementary Figure 6:

Genotype verification of the control-YFP strain

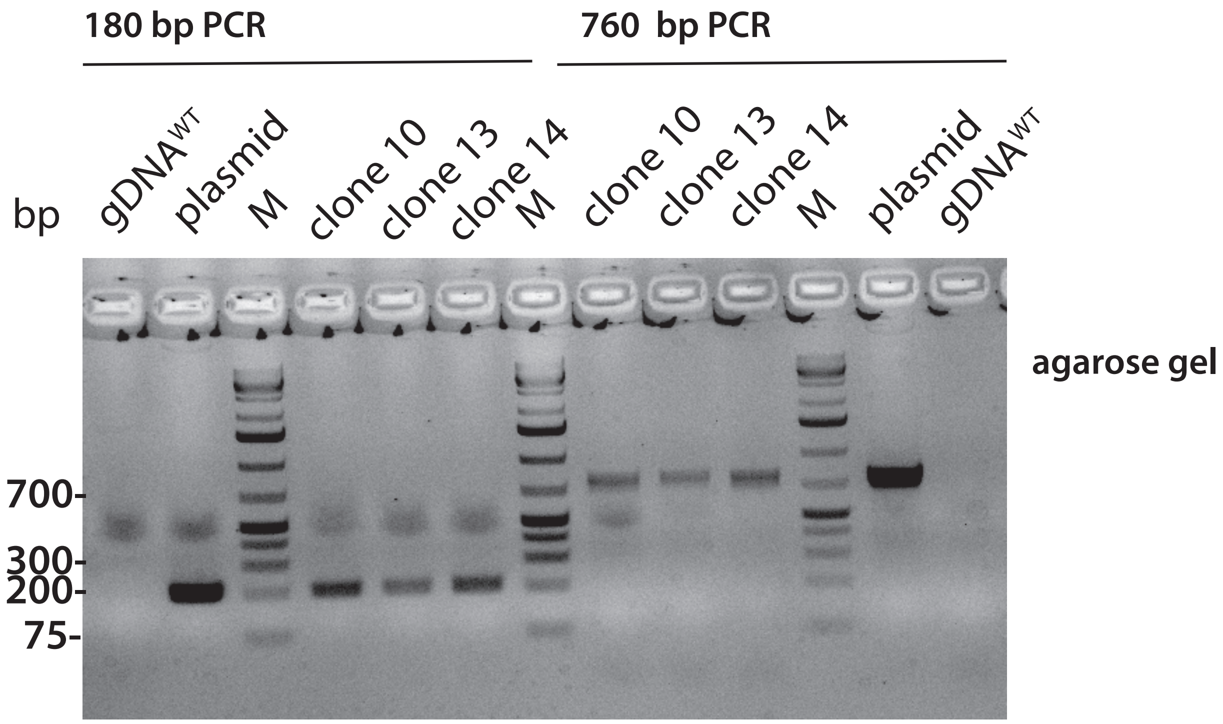

Representative clone 10:

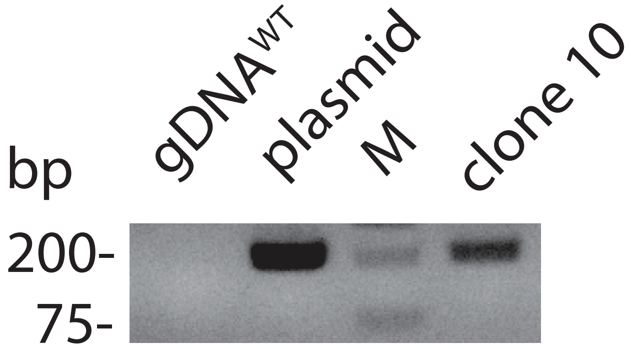

Supplementary Figure 4: unprocessed data related to Supplementary Figure 3
