# Supplementary material for: Identification and characterization of sugar-regulated promoters in Chaetomium thermophilum
Source: BMC Biotechnol. 2023 Jul 8;23:19. doi: 10.1186/s12896-023-00791-9 (PMC10329369; doi:10.1186/s12896-023-00791-9)
Supplement: Supplementary file 7 — Additional file 7. Supplementary Figure 7. unprocessed data related to Figure 4. [file 12896_2023_791_MOESM7_ESM.pdf]

Supplementary Figure 7: page 1/2

**PCBP-YFP induction in glucose and xylose media: anti-YFP blotting**

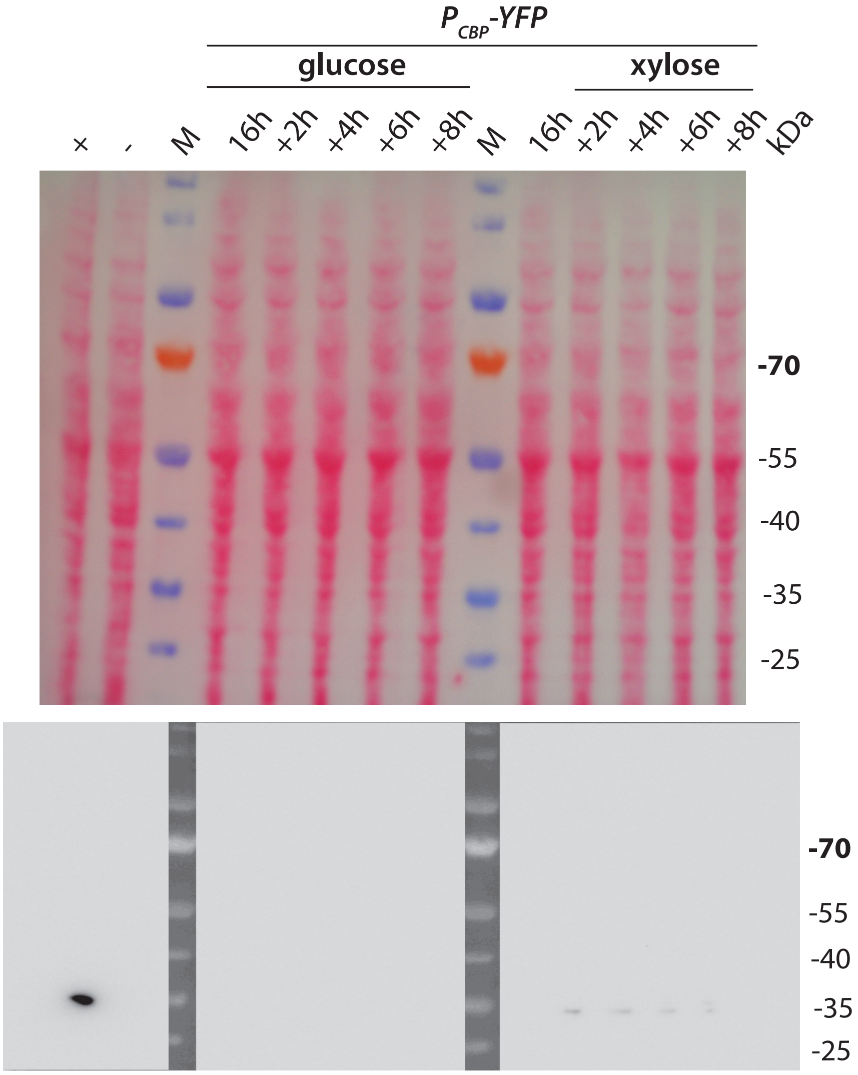


5 min Exposure time

+ = PXYL-YFP (8h xylose induced)
- = Control-YFP (8h xylose induced)

**Supplementary Figure 7:** unprocessed data related to Figure 4

Supplementary Figure 7: page 2/2

**Pxyl-YFP and PxylDH-YFP induction in glucose and xylose media: anti-YFP blotting**

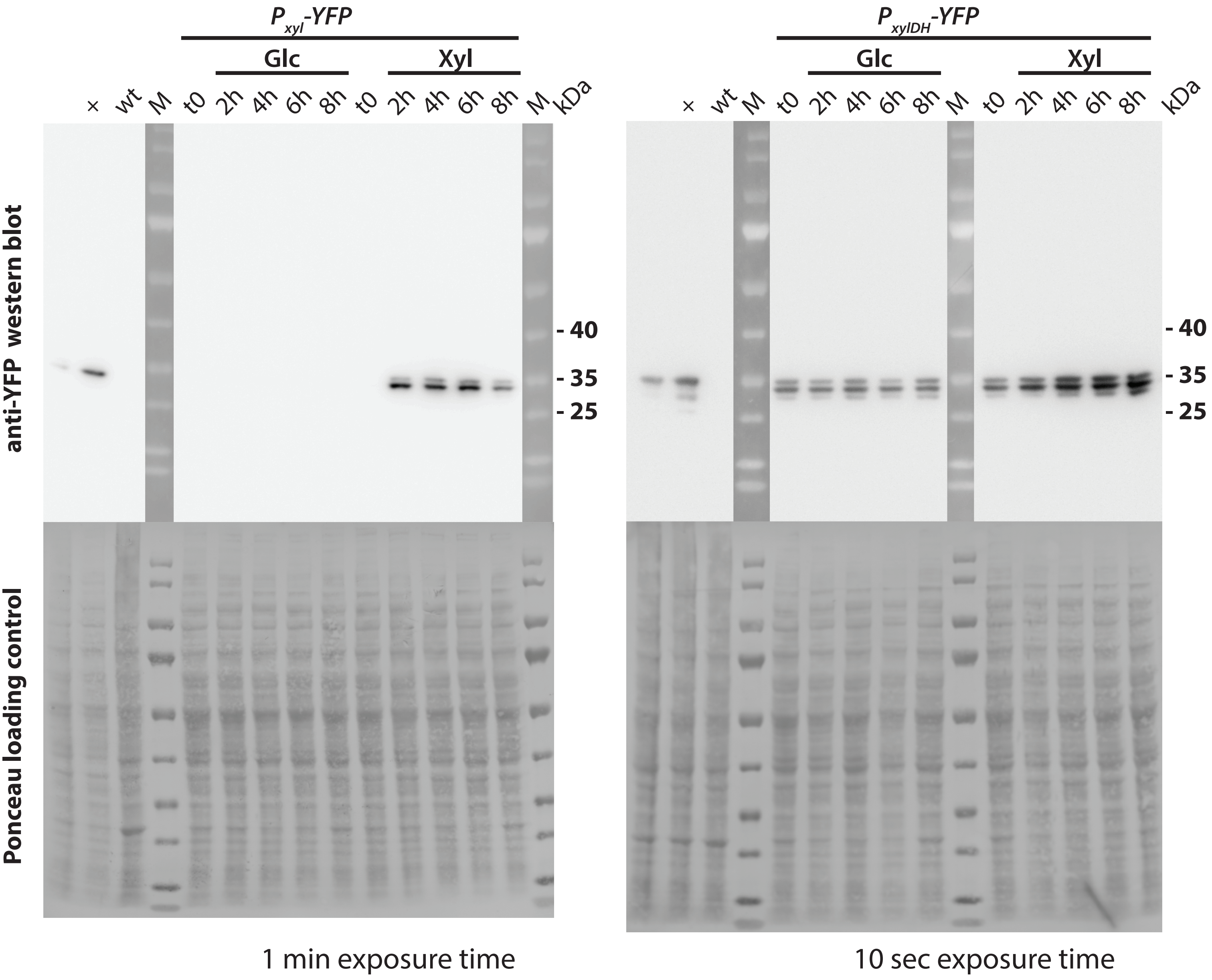


**Supplementary Figure 7:** unprocessed data related to Figure 4
