# Supplementary material for: Identification and characterization of sugar-regulated promoters in Chaetomium thermophilum
Source: BMC Biotechnol. 2023 Jul 8;23:19. doi: 10.1186/s12896-023-00791-9 (PMC10329369; doi:10.1186/s12896-023-00791-9)
Supplement: Supplementary file 9 — Additional file 9. Supplementary Figure 9. unprocessed data related to Supplementary Figure 4. [file 12896_2023_791_MOESM9_ESM.pdf]

Supplementary Figure 9:

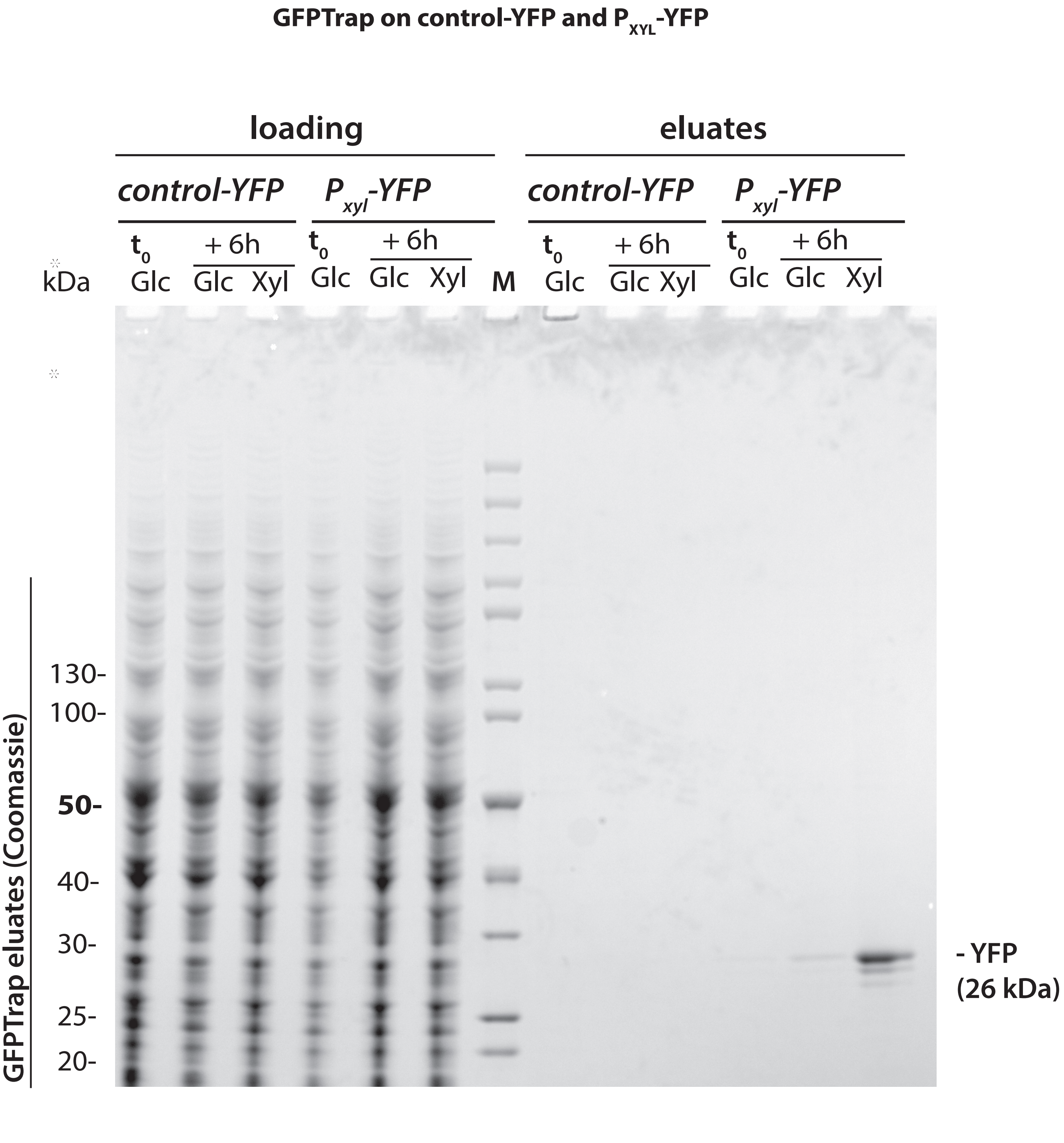

Supplementary Figure 9: unprocessed data related to Supplementary Figure 4
