# Supplementary material for: Identification and characterization of sugar-regulated promoters in Chaetomium thermophilum
Source: BMC Biotechnol. 2023 Jul 8;23:19. doi: 10.1186/s12896-023-00791-9 (PMC10329369; doi:10.1186/s12896-023-00791-9)
Supplement: Supplementary file 10 — Additional file 10. Supplementary Figure 10. unprocessed data related to Supplementary Figure 5. [file 12896_2023_791_MOESM10_ESM.pdf]

Supplementary Figure 10:

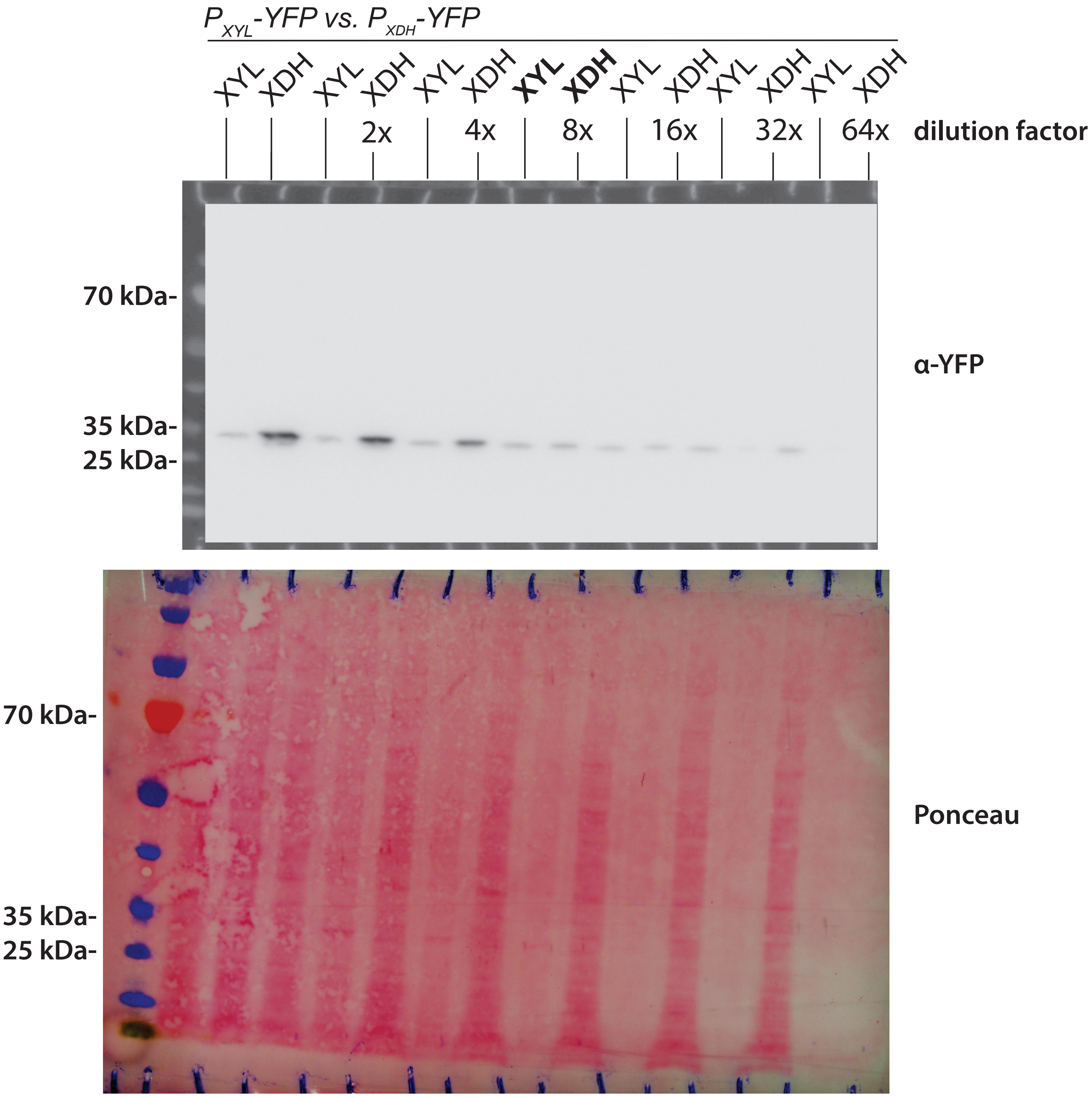

Supplementary Figure 10: unprocessed data related to Supplementary Figure 5
